# Review of Recent (2015–2024) Popular Entropy Definitions Applied to Physiological Signals

**DOI:** 10.3390/e27090983

**Published:** 2025-09-20

**Authors:** Dimitrios Platakis, George Manis

**Affiliations:** Department of Computer Science and Engineering, University of Ioannina, 45110 Ioannina, Greece; d.platakis@uoi.gr

**Keywords:** review, entropy, biomedical engineering, physiological signals, Dispersion Entropy, Bubble Entropy, Distribution Entropy, Phase Entropy, Slope Entropy

## Abstract

Entropy estimation is widely used in time series analysis, particularly in the field of Biomedical Engineering. It plays a key role in analyzing a wide range of physiological signals and serves as a measure of signal complexity, which reflects the complexity of the underlying system. The widespread adoption of entropy in research has led to numerous entropy definitions, with Approximate Entropy and Sample Entropy being among the most widely used. Over the past decade, the field has remained highly active, with a significant number of new entropy definitions being proposed, some inspired by Approximate and Sample Entropy, some by Permutation entropy, while others followed their own course of thought. In this paper, we review and compare the most prominent entropy definitions that have appeared in the last decade (2015–2024). We performed the search on 20 December 2024. We adopt the PRISMA methodology for this purpose, a widely accepted standard for conducting systematic literature reviews. With the included articles, we present statistical results on the number of citations for each method and the application domains in which they have been used. We also conducted a thorough review of the selected articles, documenting for each paper which definition has been employed and on which physiological signal it has been applied.

## 1. Introduction

Entropy is a measure of uncertainty, randomness, or disorder. In thermodynamics, it quantifies the unavailability of a system to perform useful work. In information theory, it expresses the space we need to describe a system, while in time series analysis, it serves as a measure of complexity.

The word entropy has its origins in ancient Greek. It is a compound word consisting of two ancient Greek words: “ἐν” (en), meaning “in” or “within”, and “τροπή” (tropē), meaning “turn” or “transformation”. The term first appeared in physics by the German scientist Rudolf Clausius in 1865 [1] (Clausius’s own wording: “I propose to call the magnitude S the entropy of the body, from the Greek word τροπή, transformation”).

In 1948, Shannon introduced, in a groundbreaking paper [2], the concept of entropy in the context of information theory. According to Shannon’s definition, a message with certainty carries no new information, and a completely uncertain message carries the maximum amount of information. This was expressed by the famous formula:

(1) $$H\left(X\right)=-\sum_{i=1}^{n}p_{i}\log_{2}p_{i},$$

where $p_{i}$ is the probability of a message $x_{i}$ to appear, expressed in bits. Another famous paper was published in 1991 [3], proposing Approximate Entropy, an entropy definition which embeds the time series into an *m*-dimensional space. The entropy is computed based on the probability of the distance between two vectors in this space being smaller than a given threshold. In 2002, Sample Entropy [4,5] was introduced as a variation of Approximate Entropy, claiming higher reliability and lower bias in real-world applications, due to the exclusion of self-matches, among other modifications. In practice today, Sample Entropy is used more frequently than Approximate Entropy and is the most popular entropy definition among those exploiting *m*-dimensional space embedding. We should also include in this short paragraph Permutation entropy [6], an entropy definition which follows a different approach: the time series is again embedded in an *m*-dimensional space, but entropy is estimated based on the diversity of sorting patterns. Specifically, each vector in the *m*-dimensional space is sorted, and the distribution of these sorting patterns determines the entropy. Permutation entropy was introduced in 2002 and gave a different perspective to entropy estimation.

Beyond the aforementioned definitions, numerous other entropy measures have been proposed in the literature. Interesting entropy definitions have been recently reviewed and compared in [7]. In this paper, we review and compare the impact on the research community of all recent entropy definitions proposed over the last decade (2015–2024). An initial literature review, prior to the systematic one, showed that the most common field of application was physiological signals; therefore, we chose this field as our focus. We then adopted a systematic methodology for the review, aiming to conduct the research as broadly and impartially as possible. Fourteen definitions will be examined, based on the PRISMA methodology [8]. In this methodology, queries are applied to a literature database to identify candidate papers. After a filtering process, the selected papers are reviewed. We focus on Biomedical Engineering problems; however, to limit the number of papers and maintain a systematic review process, we concentrate on physiological signals, the most popular application field for entropy. We examined the following signals: ECG/HRV (Electrocardiogram/Heart Rate Variability), EEG (Electroencephalogram), PPG (Photoplethysmogram), EHG (Electrohysterogram), CTG (Cardiotocogram), and EMG (Electromyogram).

The rest of this paper is structured as follows: In Section 2, the definitions of the examined methods are described. The PRISMA methodology is outlined in Section 3. The metadata resulting from this process is presented in tables and discussed in Section 4. The articles retained after the systematic selection are grouped according to the entropy definitions and the signals analyzed, and are reviewed in Section 5. Before the final section, Section 7, which summarizes the conclusions of the review, Section 6 intrigues the reader with an interesting discussion.

## 2. Recent Entropy Definitions

The following section provides a brief yet comprehensive overview of the definitions under examination. The definitions are presented concisely, yet with sufficient detail to ensure clarity and completeness. Additionally, we provide details on how the researchers who proposed the definition have applied it, including the parameters used and the lengths of the analyzed signals.

We use the notation $X=x_{1},x_{2},\ldots ,x_{N}$ for the original time series of size *N* and $X^{m}=x_{1}^{m},x_{2}^{m},\ldots ,x_{N-m+1}^{m}$ for the time series of vectors in the embedding space with dimension *m*. For clarity, we have gathered all the symbols we used in Table 1.

Fourteen definitions of entropy will be presented, each proposed within the decade (2015–2024). Table 2 provides us with information about the course of thought each entropy definition follows.

### 2.1. Distance-Based Entropy Definitions

Several of the examined definitions were greatly inspired by the well-established entropy definitions, Approximate and Sample Entropy, often introducing refinements to overcome their known limitations. In this subsection we gather and present entropy definitions that primarily focus on measuring distances within the original embedded $X^{m}$ time series.

#### 2.1.1. Range Entropy

Range entropy (*RangEn*) was proposed by Amir et al. in 2018 [9] as a modification of Approximate and Sample Entropy. It replaces the Chebyshev distance between $x_{i}^{m}$ and $x_{j}^{m}$ with the following metric (symbolized in the original paper as $d_{range}\left(x_{i}^{m},x_{j}^{m}\right)$):

(2) $$d_{i,j}^{m}=\frac{\max_{k}|x_{i+k}-x_{j+k}|-\min_{k}\left|x_{i+k}-x_{j+k}\right|}{\max_{k}|x_{i+k}-x_{j+k}|+\min_{k}\left|x_{i+k}-x_{j+k}\right|},\;\;\;1\leq k\leq m.$$

Two definitions of entropy $RangEn_{A}$ and $RangEn_{B}$ are proposed, based on Approximate Entropy and on Sample Entropy. $RangEn_{A}$ and $RangEn_{B}$ were compared with Approximate and Sample Entropy, presenting a smaller standard deviation for various signal lengths (N=50 up to N=1000), with embedding dimension m=2 and threshold distance r=0.2 [9].

#### 2.1.2. Cosine Similarity Entropy

Cosine Similarity (*CosEn*) entropy was introduced by Theerasak et al. in 2017 [10]. It replaces (a) the Chebyshev distance, originally adopted by Sample Entropy, with the Angular Distance:

(3) AngDisti,jm=(1π)cos−1xim·xjm||xim||||xjm||,

and (b) the standard conditional probability with Shannon entropy, (originally symbolized $AngDist_{i,j}^{m}=MMa_{i,j}^{\left(m\right)}\;/\;\pi$, where $a_{i,j}^{\left(m\right)}=cos^{-1}\left(CosSim_{i,j}^{\left(m\right)}\right)$ and $CosSim_{i,j}^{\left(m\right)}=MMx_{i}^{m}\cdot x_{j}^{m}\;/\;\left|\right|x_{i}^{m}\left||\;|\right|x_{j}^{m}\left|\right|$).

Since *CosEn* employs Shannon entropy, the authors expected the method to exhibit the same characteristics. Maximum values of Cosine Similarity entropy are presented for r=0.5. The proposed values for *r* are between 0.05 and 0.2, or 0.5 and 1. The authors proposed m=2…5, r=0.05…0.2 and minimum N=100 for WGN and 1f noise or N=700 for first- or second-order Auto-Regressive processes [10].

#### 2.1.3. Diversity Entropy

Diversity entropy (*DivEn*) was introduced by Xianzhi et al. in 2021 [11] to address certain inconsistencies present in Sample Entropy, Fuzzy entropy [12], and Permutation entropy. It computes the similarity between adjacent vectors $x_{i}^{m},x_{j}^{m}$ (originally symbolized as $y_{i}\left(m\right),y_{j}\left(m\right)$) using the Cosine Similarity (originally symbolized as $d(y_{i}\left(m\right),y_{j}\left(m\right))$:

(4) $$d_{i,j}^{m}=\frac{x_{i}^{m}\cdot x_{j}^{m}}{|x_{i}^{m}\left|\right|x_{j}^{m}|}.$$

The Cosine Similarity is ranged from −1 to 1. All those distances are mapped onto *k* bins, $B_{k}$. The probability $p_{k}$ of a distance falling within $B_{k}$ is defined. Diversity entropy is computed by Shannon entropy on the distribution of $p_{k}$.

Diversity entropy was originally proposed as a measure to diagnose fault diagnosis in rotating machinery, where it reports low entropy values for deterministic series and higher values for chaotic ones using m=4 and k=100 [11].

#### 2.1.4. Distribution Entropy

Distribution Entropy (*DistEn*) was proposed by Li et al. in 2015 [13]. The time series is again embedded in *m*-dimensional space. For each vector $x_{i}^{m}$ (originally symbolized as x(i)), the maximum distance $d_{i,j}^{m}$ from all other vectors is computed. These distances are binned into *M* equal-sized bins. The empirical probability function is calculated:

(5) $$p_{t,m,M}=\frac{\#\mathrm{elements}\;\mathrm{in}\;\mathrm{bin}t}{\mathrm{total}\#\mathrm{elements}},$$

where t=1,2,…,M. Distribution Entropy is reported as

(6) $$DistEn_{m,M}=-\frac{1}{\log_{2}M}\sum_{t=1}^{M}p_{t,m,M}\log_{2}p_{t,m,M},$$

where the unit of the chosen base $\log_{2}$ is bits.

In [13], using five types of chaotic series and stochastic processes, Distribution Entropy exhibited increased stability for N=50,…,200 compared to Sample and Fuzzy entropy. It presented a remarkable stability for M=512,…,1024. Using m=1,…,10, Distribution Entropy was stable both for the average levels and the standard deviations.

### 2.2. Symbolic and Ordinal Pattern-Based Entropy Definitions

Alongside Approximate and Sample Entropy, Permutation entropy has emerged as one of the widely used entropy definitions that also embeds the original time series *X* into $X^{m}$. However, it analyzes the distribution of the ordinal patterns of the vectorized time series $X^{m}$. This different approach to quantify irregularity in a time series inspired researchers to extend Permutation entropy to initially map the original time series, followed by an investigation of the ordinal patterns. In this section we will present the entropy definitions with a similar concept.

#### 2.2.1. Increment Entropy

Inspired by symbolic dynamics and Permutation entropy, Increment entropy (*IncrEn*) [14] takes into consideration the amplitude of the time series and the difference in successive samples.

Given the original time series *X*, the increment time series $V=v_{1},v_{2},\ldots ,v_{N-1}$ is constructed, where $v_{i}=x_{i+1}-x_{i}$. The time series *V* is embedded into the *m*-dimensional space, giving the series of vectors $V^{m}=v_{1}^{m},v_{2}^{m},\ldots ,v_{N-m}^{m}$.

Each element of each vector is mapped onto a two-letter word. The first letter is the sign *s* and the second is the magnitude *q*, based on the quantifying resolution parameter *R*. The series $U^{m}=u_{1}^{m},u_{2}^{m},\ldots u_{N-m}^{m}$ consists of the vectors $u_{i}^{m}=\overset{m-1}{\underset{j=0}{⋃}}s_{i+j}q_{i+j}$ (originally symbolized as $\left\{w_{i},\;1\leq i\leq N-m\right\}$). By computing the number of instances of $u_{i}^{m}$, and the probability of appearance, $p(u_{i}^{m})$, Increment entropy is reported as

$$IncrEn\left(m\right)=-\frac{1}{m-1}\sum_{i=1}^{{\left(2q+1\right)}^{m}}p\left(u_{i}^{m}\right)\log_{2}p\left(u_{i}^{m}\right),$$

where entropy is given in bits.

The authors in [14] propose m=2,…,5 and a quantifying resolution of R≤4. However, further research [15] showed that in real-life applications, *IncrEn*’s parameters m,R,N become sensitive for short signals N≤500, reaching a stabilized state after N=1000. Short signals’ optimal parameters are 2≤m≤6 and 2≤R≤8; specifically, *m* and *R* should take values between 2 and 4.

#### 2.2.2. Dispersion Entropy

In 2016, Rostaghi and Azami [16] proposed Dispersion Entropy (*DispEn*), an entropy definition that combines the order of the samples and their values and also employs the time delay for the embedding process.

Starting with the given time series *X*, a new mapped series $U=u_{1},u_{2},\ldots ,u_{N}$ is produced, labeled from 1 to *c*. The series is embedded in the *m*-dimensional space with time delay *d*:

(7) $$U^{m,c}=u_{1}^{m,c},u_{2}^{m,c},\ldots ,u_{\left(N-m+1\right)}^{m,c},\;\;\;\;u_{i}^{m,c}=\left(u_{i}^{c},u_{i+d}^{c},\ldots ,u_{i+(m-1)d}^{c}\right).$$

Originally, $u_{i}^{m,c}$ is symbolized as $z_{i}^{m,c}=\left\{z_{i}^{c},z_{i+d}^{c},\ldots ,z_{i+(m-1)d}^{c}\right\}$. The ordinal pattern $\pi_{i}^{m}=\left(u_{i},u_{i+1},\ldots ,u_{i+m-1}\right)$ (originally symbolized as $\pi_{v_{0}v_{1}\ldots v_{m-1}}$, where $z_{i}^{c}=v_{0},\;z_{i+d}^{c}=v_{1},\ldots ,z_{i+(m-1)d}^{c}=v_{m-1}$) is calculated and stored. The number of possible dispersion patterns for each $u_{i}^{m}$ is $c^{m}$. For each pattern $\pi_{i}^{m}$ the probability of appearance $p(\pi_{i}^{m})$ is computed. The Shannon entropy of the probability of the appearances reports the Dispersion Entropy.

(8) $$DispEn_{X,m,c,d}=-\sum_{i=1}^{c^{m}}p\left(\pi_{i}^{m}\right)\ln p\left(\pi_{i}^{m}\right).$$

The entropy is expressed in nats. Normalized DispEn is calculated as $DispEn_{normalized}=MMDispEn\;/\;\ln c^{m}$, where $\ln c^{m}$ is the largest DispEn value.

For labeling the original signal *X*, both linear and nonlinear methods can be used [16], with normal cumulative distribution function (NCDF) showing superiority over linear mapping techniques and stabilization at the maximum value after N=1000.

#### 2.2.3. Fluctuation-Based Dispersion Entropy

Inspired by Dispersion Entropy, Hamed et al. proposed Fluctuation-based Dispersion Entropy (*FDispEn*) in 2018 [17]. In Dispersion Entropy, the estimation of each element of the embedded time series $X_{i}^{m}$ has *c* possible values; thus, each vector has $c^{m}$ possible patterns. Fluctuation-based Dispersion Entropy considers the differences between adjacent elements of dispersion patterns, leading to vectors of size m−1 with ${\left(2c-1\right)}^{m}$ possible states. A normalized version of *FDispEn* is given by the formula $\frac{FDispEn}{ln{\left(2c-1\right)}^{m-1}}$.

#### 2.2.4. Slope Entropy

Slope entropy (*SlopEn*) was introduced by David Cuesta-Frau in 2019 [18]. It was inspired by Permutation entropy, a definition where the amplitude information is ignored. The time series is embedded into an *m*-dimensional space, giving the series $X^{m}$. The difference between two consecutive elements of the vector $x_{i}^{m}$ is computed, resulting in the series $D^{m-1}\;\;=\;d_{1}^{m-1},d_{2}^{m-1},\ldots ,d_{N-m+1}^{m-1}$.

**Figure 1:**
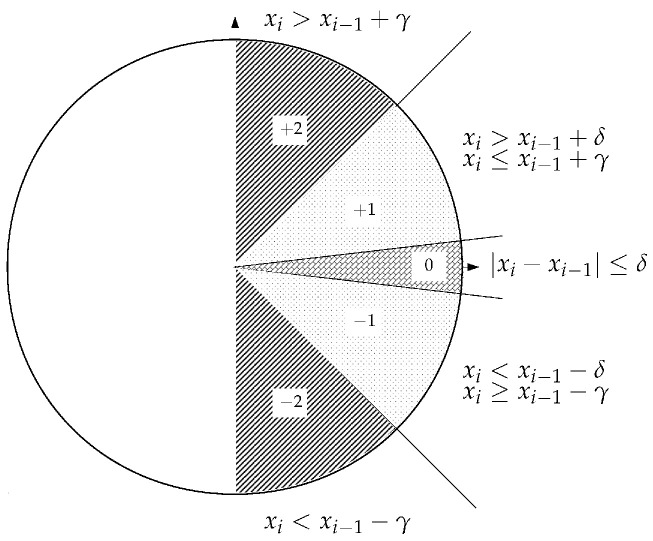
Schematic illustration of the mapping method. Figure from the “Entropy” journal [18].

Each element of each vector $d_{i}^{m-1}$ is mapped onto one of the possible five symbols +2,+1,0,−1,−2, as shown in Figure 1. The sizes of the sectors are described by the two parameters, γ and δ. The generated series $U^{m-1}$ consists of the vectors $u_{i}^{m-1}$, where each element is a symbol. The Shannon entropy of the distribution function $p_{i}$, i.e., the probability of a vector of symbols $u_{i}^{m-1}$ (originally symbolized as $\psi_{i}^{m}$) to appear, defines the Slope entropy. Slope entropy managed to show great discriminating capabilities for low *N* values (250 and 500) and m=3,…,8. The recommended γ and δ parameters are γ≈1,2 and δ close to zero [18]. A simplified Slope entropy definition is proposed where δ is discarded along with the zero symbol in the mapping procedure [19] or an asymmetry regarding the γ parameter [20].

#### 2.2.5. Symbolic Dynamic Entropy

Symbolic Dynamic entropy was proposed by Li et al. [21] and is based on the state transition probability of the possible state patterns. It was initially inspired by a similar entropy definition [22] and addressed its shortcomings. The original time series *X* is transformed using either Uniform Partitioning or Maximum Entropy Partitioning into a new series $U=u_{1},u_{2},\ldots ,u_{N}$ (originally symbolized as *Z*) consisting of *c* (originally symbolized as ϵ) number of symbols. Then, the new series *U* is embedded in *m*-dimensional space with delay *d* (originally symbolized as λ):

(9) $$U^{m,c}=u_{1}^{m,c},u_{2}^{m,c},\ldots ,u_{\left(N-m+1\right)}^{m,c},\;\;\;\;u_{i}^{m,c}=\left(u_{i}^{c},u_{i+d}^{c},\ldots ,u_{i+(m-1)d}^{c}\right).$$

Symbolic Dynamic Entropy is given by

$$sde\left(X,m,d,c\right)=-\sum_{a=1}^{c^{m}}p\left(u_{a}^{c,m,d}\right)\cdot \ln p\left(u_{a}^{c,m,d}\right)-\sum_{a=1}^{c^{m}}\sum_{b=1}^{c}p\left(u_{a}^{c,m,d}\right)\cdot \ln\left(p\left(u_{a}^{c,m,d}\right)\cdot p\left(\sigma_{b}|u_{a}^{c,m,d}\right)\right),$$

expressed in nats, with $p(u_{a}^{c,m,d})$ expressing the probability that the $u_{a}^{c,m,d}$ pattern will appear and $p(\sigma_{b}|u_{a}^{c,m,d})$ expressing the probability that the $\sigma_{b}$ symbol will appear given that the $u_{a}^{c,m,d}$ pattern has appeared. Employing a normalization factor, Symbolic Dynamic Entropy is reported as

$$SDE\left(X,m,d,c\right)=sde\left(X,m,d,c\right)/lnc^{m+1}.$$

In order to find the optimal parameters, researchers in [21] propose an algorithm based on the Average Euclidean Distance (AED).

### 2.3. Complexity Estimation Based on Sorting Effort

The ordinal patterns of Permutation entropy are obtained by sorting each embedded vector $x_{i}^{m}$, a procedure that is also employed by Bubble Entropy to extract valuable information about the series regularity.

#### Bubble Entropy

Bubble Entropy (*BubbleEn*) was introduced in 2017 [23] as an entropy definition “almost free of parameters”. It was designed to free researchers from the need to estimate parameters. Instead of using the distribution of the sorting patterns to estimate entropy, Bubble Entropy used the distribution of the tasks spent to perform the sorting of the elements of each $x_{i}^{m}$, giving a physical meaning to what is distributed, since task means energy, a physical quantity very closely related to entropy. In addition, Bubble Entropy reduces the number of possible states in the distribution, leading to better-balanced distributions. The method reports the difference in entropy between spaces of size m+1 and *m*. The description of the method is as follows.

The elements in each vector $x_{i}^{m}$ are sorted, and the number of swaps $s_{i}$ performed by the bubble sort algorithm is what counts as a task. A new time series, the series of the sorting tasks, is formed:

(10) $$s_{i}=s_{1},s_{2},\ldots ,s_{N-m+1}.$$

The probability mass function $p_{i}$ of having *i* swaps is used to evaluate the second-order Rényi entropy, expressed in bits:

(11) Hswapsm=−log2∑i=0m2pi2.

Bubble Entropy is the normalized difference in the entropy of the sorting effort (swaps) required to sort vectors of length m+1 and *m*:

(12) $$BubbleEn_{m}=K^{m}\left(H_{\mathrm{swaps}}^{m+1}-H_{\mathrm{swaps}}^{m}\right),$$

where $K^{m}$ is a normalization factor. For more information on normalization factors for Bubble Entropy, as well as for the study of several theoretical issues, please see [24], where three normalization options are suggested. The first option is not to normalize, the second is normalization based on the maximum number of possible states, and the last one is normalization based on the White Gaussian Noise.

The computation cost of Bubble Entropy is quite low, leading researchers to claim that there is no need to define a restricted range of *m* values, since it is feasible to compute a broad range of *m*. In [23] researchers used m=2,…,25, with the m≥12 range exhibiting valuable discriminative information. Using a synthetic series Bubble Entropy presented stability for short-length signals. In [24,25], they computed Bubble Entropy for 2≤m≤50, and again, the range of 10<m<20 was observed as the most valuable one.

### 2.4. Multiscale and Hierarchical Definitions

The dynamics of a time series can be further explored through a scaling process, a powerful modification widely applied to many entropy definitions. Entropy of Entropy employs this approach to reveal the underlying dynamics.

#### Entropy of Entropy

In 2017, Chang Francis Hsu et al. proposed the definition of Entropy of Entropy (*EoE*) [26], a novel complexity measurement inspired by Multiscale entropy [27]. The original series *X* is divided into non-overlapping windows of size τ.

(13) $$W^{\tau }=w_{1}^{\tau },w_{2}^{\tau },\ldots ,w_{\left\lfloor N/\tau \right\rfloor }^{\tau }.$$

In the range of $\min(w_{j}^{\tau })$ and $\max(w_{j}^{\tau })$ elements, where 1≤j≤⌊N/τ⌋, the window is divided into *s* slices of equal size. The probability $p_{i}$ of a sample $x_{i}$ to be in slice *k*, where $1\leq k\leq s_{1}$, is computed as

(14) $$p_{j,k}=\frac{\#x_{i}\;\mathrm{over}\;w_{j}^{\tau }\;\mathrm{in}\;\mathrm{state}\;k}{\tau }.$$

Next, the Shannon entropy of each $w_{i}^{\tau }$ is computed:

(15) $$y_{i}^{\tau }=-\sum_{k=1}^{s_{1}}p_{i,k}\ln p_{i,k}.$$

The number of states *L*, where 1≤L≤all_states (originally all_states are symbolized as $s_{2}$), for each $y_{j}^{\tau }$ is both finite and dependent on τ. The probability $p_{L}$ for the state $y_{j}^{\tau }$ over the generated series $Y^{\tau }=y_{1}^{\tau },y_{2}^{\tau },\ldots ,y_{MMN\;/\;\tau }$ to occur in state *L* is obtained by the following form:

(16) $$p_{L}=\frac{\mathrm{total}\;\mathrm{number}\;\mathrm{of}\;y_{j}^{\tau }\;\mathrm{over}\;Y^{\tau }\;\mathrm{in}\;\mathrm{level}\;L}{N/\tau }.$$

Entropy of Entropy is computed for all finite possible states:

(17) $$EoE_{\tau ,s}=-\sum_{L}^{\mathrm{all}\_\mathrm{states}}p_{L}\ln p_{L}.$$

In [26] the authors who proposed the method used Entropy of Entropy to discriminate between two pathological groups. The best results were reported with τ≤5 and for 55 slices. When compared with Multiscale entropy, EoE performed sufficiently for a short time series.

### 2.5. Geometric or Phase-Space Definitions

Given a time series *X*, a Poincaré-Lorenz plot represents *X* in a two-dimensional space, with the *x*-axis representing the current $x_{i}$ state and the y-axis the next one, $x_{i+1}$. A diagonal line separates points that declare acceleration from points that declare deceleration. Thus, we can visually extract information about the dynamics of the series. Phase and Gridded Distribution Entropy adapts Poincaré-Lorenz plots in their definition to measure the regularity of a series.

#### 2.5.1. Phase Entropy

Phase entropy (*PhEn*) was introduced by Ashish et al. in 2019 [28], a definition based on the Poincaré-Lorenz plot. A second-order difference Poincaré-Lorenz plot is also informative, where the *x*-axis represents the difference $x_{i+1}-x_{i}$ and the y-axis the difference $x_{i+2}-x_{i+1}$. The plot is divided into four quadrants. One of the quadrants reveals two consecutive accelerations. Another one reveals two consecutive decelerations. There is one quadrant revealing one acceleration followed by one deceleration, and one quadrant for a deceleration followed by one acceleration.

For each point the slope angle is calculated:

(18) $$\theta_{i}=\tan^{-1}\frac{x_{i+2}-x_{i+1}}{x_{i+1}-x_{i}}.$$

In general, the plot can be divided into *k* instead of four sectors. The function $p_{i}$ is formulated by the slope angles:

(19) $$p_{i,k}=\frac{\sum_{j\;\mathrm{in}\;\mathrm{sector}\;i}\;\theta_{j}}{\sum_{j\;\mathrm{in}\;\mathrm{all}\;\mathrm{sectors}}\;\;\;\theta_{j}}.$$

Finally, the entropy is estimated in bits by

(20) $$PhEn_{k}=-\frac{1}{\log_{2}k}\sum_{i=1}^{k}p_{i,k}\log_{2}p_{i,k}.$$

Researchers in [28] propose k≥16, or a value dividable by four. Phase entropy showed a stability on generated White Signal Noise of various lengths, starting with N=100 up to *N* = 50,000.

#### 2.5.2. Gridded Distribution Entropy

Gridded Distribution Entropy (*GDEn*) was introduced by Chang Yan et al. in 2019 [29]. The time series *X* is filtered and scaled between 0 and 1. Then, like Phase entropy, a Poincaré plot is generated, which is divided into n∗n blocks, yielding a gridded Poincaré plot. Let us assume that each block contains a finite number of $\beta_{k}$ points, where k=1,2,…,n∗n. The Gridded Distribution Rate is computed:

(21) $$GDR=\frac{\alpha }{n*n},$$

where α is the number of blocks with at least one point in it. Gridded Distribution Entropy is computed by the Shannon entropy of $p_{k}$, where

(22) $$p_{k}=\frac{\beta_{k}}{N-1}.$$

In [29], the method becomes stable when the *n* parameter is greater than 80.

### 2.6. Pattern-Detection Definitions

An innovative course of thought in regularity analysis is introduced by Attention entropy, where the investigation is based upon some key patterns present in the time series.

#### Attention Entropy

Attention entropy (*AttEn*) was proposed by Jiawei Yang et al. [30] in 2023. The time series is not embedded into an *m*-dimensional space but is based on key pattern detection. The number of samples between key patterns gives a series on which Shannon entropy is computed. In [30] the local minimum and local maximum are proposed as key patterns. Attention entropy consistently discriminated HRV signals [30], with lengths starting from N=100, up to *N* = 10,000.

## 3. Methodology

We followed the PRISMA (Preferred Reporting Items for Systematic Reviews and Meta-Analyses) protocol [8] to review the published literature systematically. Articles, initially collected from the Scopus article database, were screened to exclude publications not relevant to the purpose of our review. Then, we checked the eligibility of each article to further filter those we were going to review. The following questions drove us to the initial selection:

- Does it propose a new entropy definition?
- Has it been published during the last decade (2015–2024)?
- Has the definition been used to analyze physiological signals (ECG/HRV, CTG, EEG, PPG, EHG, EMG)?

We used the Scopus article database to collect any articles that

- Refer to any entropy definition investigating or including the name of the entropy definition in the title, abstract, or paper keywords.
- Are related at any point with either EEG, ECG/HRV, CTG, EMG, EHG, or PPG, or the word “Biomedical”.
- Belong to the “Computer Science and Engineering” field, as the most relevant available superset of Biomedical Engineering.

The queries that were used to initially collect and count the articles for each entropy definition were structured as follows:

(TITLE-ABS-KEY ("entropy definition")) AND
(ALL ("EEG" OR "ECG" OR "HRV" OR "PPG" OR "Biomedical"
OR "CTG" OR "EHG" OR "EMG"))
AND PUBYEAR > 2014 AND PUBYEAR < 2025 AND
(LIMIT-TO (SUBJAREA,"ENGI") OR LIMIT-TO (SUBJAREA,"COMP"))

The search was performed on 20 December 2024. All publications and their citations dated before that point were considered. We first collected all articles returned as relevant by the database query. Since some articles appeared more than once, we removed the duplicates to ensure only unique entries remained. In the next step, we kept only those articles belonging to the Biomedical field, studying physiological signals. Next, from the retained articles, we further selected only those employing at least one of the recent entropy definitions examined in this review. The remaining articles formed the basis for our statistical analysis and the following discussion.

## 4. Search, Selection, and Descriptive Statistics

With the above methodology and with the applied search criteria, we initially collected 516 articles. By removing duplicates, the number of articles was reduced to 447.

Through the screening process, we limited the articles to those belonging to the Biomedical Engineering field and, specifically, those examining physiological signals. We read the abstracts of all 447 papers and selected 213 articles as relevant. Apart from the Biomedical Engineering field, two other scientific fields were also very popular: “fault diagnosis” and “marine”.

In the final stage, we read the whole text of every paper and kept only those employing the examined entropy definitions. We have to note that we included papers applying the base entropy definitions (i.e., as they were initially proposed) and not subsequent modifications. Out of the 213 articles, 92 of them passed this final criterion.

We used a systematic approach in both the selection of entropy definitions and papers reviewed. This limits the possibility to exclude one or more definitions. A review of the literature before the systematic review helped in this direction.

**Figure 2:**
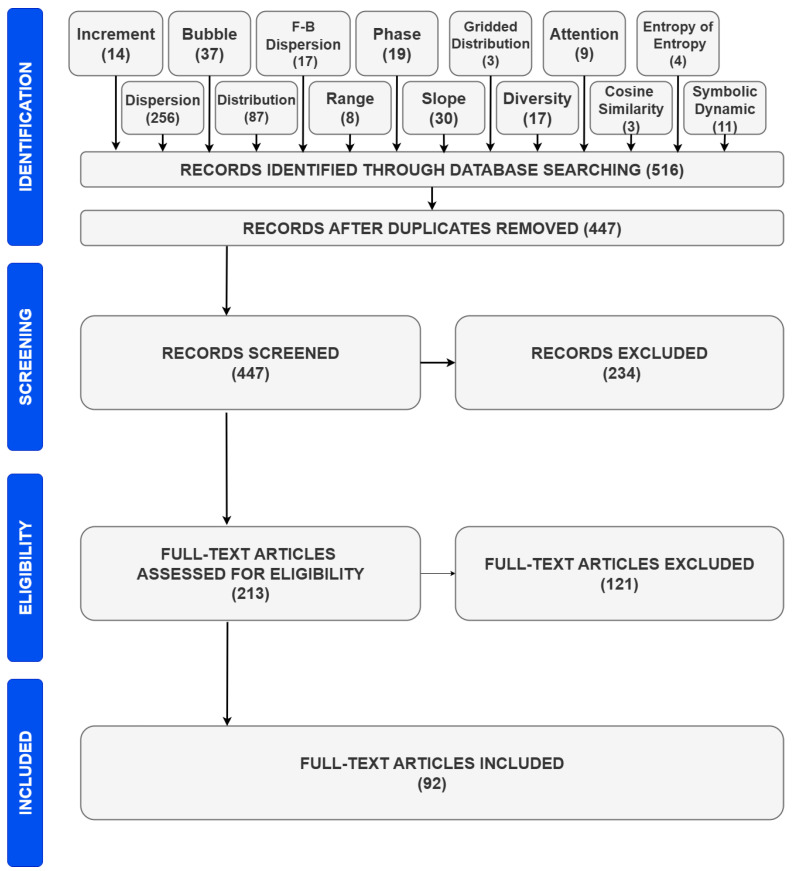
The PRISMA diagram.

In Figure 2 the filtering procedure is presented through a diagram, as suggested by the PRISMA protocol. At the top of this figure, we can see the identification phase, where all entropy definitions examined in this review are displayed. Each definition is accompanied by the number of articles initially selected as relevant. The identification phase is followed by the screening phase, the eligibility phase, and the inclusion phase.

Table 3 presents the number of articles referring to each definition. The first column indicates the total number of references identified for each definition. The following column indicates the number of articles containing the name of the entropy definition in their title, the third one in the keywords, and the last one in the abstract. We can see that Dispersion Entropy is the most mentioned one (31 references in total), followed by Bubble Entropy (25) and Distribution Entropy (22). The rest of the definitions present a slightly smaller number of citations. It should be noted that the sum of the column “As a Citation” is not 92 (the number of the examined articles), since many papers referenced more than one definition.

Table 4 shows the number of articles per examined method and physiological signal. For the same reason, as in Table 3, the summation of the columns or the rows does not give the aforementioned numbers. An interesting observation resulting from this table is that Bubble Entropy is the only entropy definition that has been used for the analysis of all examined physiological signals. It should be noted that Diversity entropy does not present any citations, since it has not been used solely as a base entropy in Biomedical signals. We included it in the review, though, for completeness, since it is a recent entropy definition.

**Figure 3:**
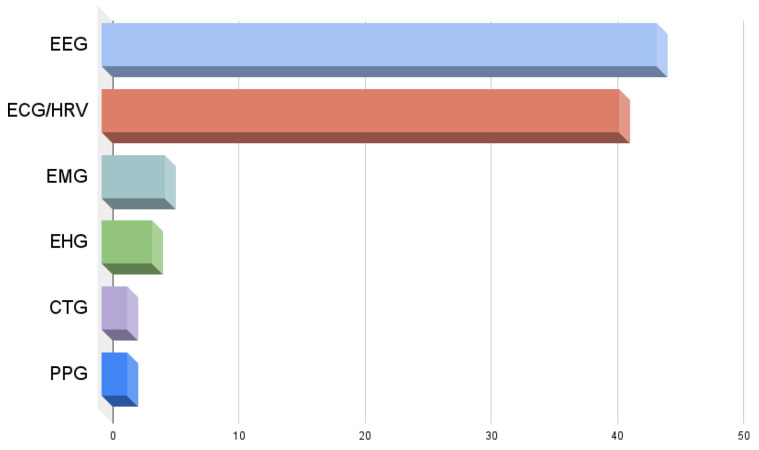
Number of applications per physiological signal.

Since, from Table 4, we cannot conclude the number of the reviewed papers for each physiological signal, we added a figure with this information. In Figure 3 we can see that the most commonly used signals are EEG (43) and ECG/HRV (41). Also included, but with a much smaller number of references, are EHG (5), EMG (5), CTG (2), and PPG (2).

**Figure 4:**
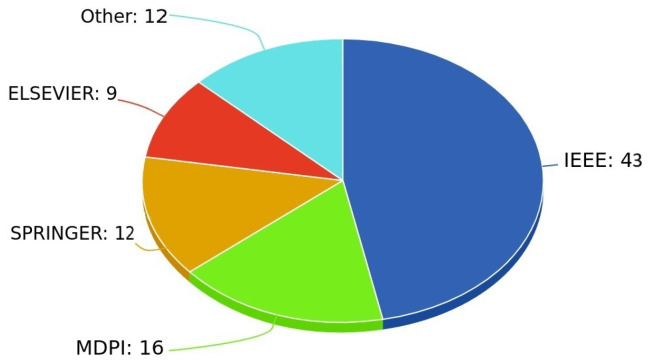
Number of articles per publisher.

Finally, Figure 4 shows the distribution of papers published in journals and conferences across different publishers. IEEE accounts for the largest share of papers (43), followed by MDPI (16, 12 of which are published in the “Entropy” journal), Springer (12), and Elsevier (9). An additional number of papers (12) can be found in various other publishers.

## 5. The Literature Review After the Systematic Article Selection

In the following, the retained articles are briefly examined by presenting the entropy definitions they used and the physiological signals on which they were applied.

### 5.1. Articles on Electroencephalogram

An EEG records electrical activity in the brain. Brain wave variability reflects fluctuations in these brain waves, which can indicate different mental states, cognitive load, or neurological health. Greater variability may suggest better brain adaptability and functioning.

Out of the 92 papers having passed the filters, 43 of them have applied the examined entropy definitions on an EEG. Seizure detection was the most popular target, while many papers detected sleep stages or emotional states.

Dispersion Entropy is applied in [16,31,32,33], to classify healthy and epileptic subjects, and in [34], to distinguish between normal, ictal, and non-ictal states. Dispersion Entropy is utilized in [35], again for the classification of healthy and epileptic subjects. Increment entropy is employed in [15,36] to categorize healthy and epileptic subjects, as well as ictal and interictal phases. Dispersion Entropy is used in [37] to categorize healthy subjects, subjects during interictal epileptic activity, and seizure attacks. In [18] Slope entropy differentiates seizure and seizure-free recordings. Slope entropy is also featured in [33], along with Dispersion, Increment, and Phase entropy. Distribution Entropy was also used to analyze ictal and interictal patients [38], to detect epileptic seizures [39], and, finally, along with FB-Dispersion Entropy, was used for seizure/non-seizure classification [40].

Sleep stage detection and sleep disorder detection are also popular subjects. Bubble Entropy is used in [41] for sleep and wake state classification and in [42] for detecting sleep spindles. Dispersion Entropy detects sleep stages in [43]. Both Bubble and Dispersion Entropy discriminate wakefulness and sleep stage in [44]. Range entropy employs statistical analysis to classify subjects into distinct states of wakefulness, drowsiness, and sleep [45]. Gridded Distribution Entropy detects and classifies sleep disorders, such as insomnia, narcolepsy, periodic leg movement, nocturnal frontal lobe epilepsy, bruxism, REM (Rapid Eye Movement) behavior disorder, and sleep-disordered breathing in [46]. In [47] Dispersion and Bubble Entropy have been computed on an EEG to discriminate four states: wake, light sleep, deep sleep, REM, and non-REM.

A significant number of papers detected emotional states through the complexity of the EEG signal. Dispersion Entropy is used for this purpose in [48,49,50,51], Increment entropy in [52], Bubble Entropy in [53], and Distribution Entropy in [54]. The detection of the stress level, as well as the valence or the arousal, is examined in [55,56] by Dispersion Entropy. Depression is detected by Distribution Entropy in [57] and alertness by Range entropy in [58].

Some fields were less popular. In [59], *FDispEn* detected eye-blinking artifacts, while *DispEn* identified different movements task [60].

Bubble Entropy with *FDispEn* discriminated subjects with mild cognitive impairment or vascular dementia and controls [61], while Bubble Entropy, along with Attention and Symbolic Dynamic entropy, is used for the same purpose in [62]. Dispersion Entropy was employed for Attention-Deficit/Hyperactivity Disorder detection, for channel identification, for classification between healthy persons and persons with Alzheimer’s disease, and along with Slope entropy, for cognitive task classification [63,64,65,66]. Bubble and Slope entropy have been utilized in speech recognition tasks [67], while *PhEn*, *GDEn*, and *CosEn* have been applied to the evaluation of brain death and coma patients [68]. Finally, Slope entropy used a single frontal EEG to predict the index of the Depth of Anesthesia [69], and Distribution Entropy for identity authentication [70].

### 5.2. Articles on Heart Signals

An ECG measures the electrical activity of the heart. Heart Rate Variability expresses the variations in time between heartbeats, which can indicate the balance between the sympathetic and parasympathetic nervous systems. Higher HRV often reflects better cardiovascular health and resilience.

This category was also proved to be popular since, out of the 92 papers that passed the filters, 41 of them have applied the examined entropy definitions on heart signals.

Distribution Entropy is the definition with the largest number of applications on heart signals among the examined methods. It has been used for arrhythmia detection [71,72,73], for congestive heart failure [13,74,75], and for Chagas disease [76]; it has also been applied to data sets with young and elderly subjects [13,73,75,77] and for coronary artery disease before and after intervention [78]. Other applications of Distribution Entropy include sleep stage detection [79], rest–walk state [80], and rest–tilt state [81] recognition. Finally, Distribution Entropy has been applied for recognizing emotions [54] and for cognitive tasks [82].

Dispersion Entropy has been used for biometric simulation [83] and for sleep stage recognition [43]. It has also been used for atrial fibrillation in a four-class detection problem [84] and to distinguish normal recordings from recordings with premature beats [85]. Finally, coronary artery disease is detected using young and elderly subjects [86], while young and elderly subjects are also studied in [16,62].

Increment entropy has been employed for stress evaluation [87,88], epilepsy detection in conjunction with an EEG [15], and sudden cardiac death prediction [89].

Bubble Entropy has been employed in congestive heart failure recognition [23,24,90] and for a tolerance to spikes study [91]. It has also been employed for discrimination between normal sinus rhythm, congestive heart failure, coronary artery disease, and sudden cardiac death [92]. Slope entropy has been used for the classification of recordings of young and elderly subjects [18]. Fluctuation-based Dispersion Entropy, introduced in [17], was originally applied to analyze rat blood pressure signals.

Entropy of Entropy was applied to analyze heart signals before and after renal artery denervation [93] and to discriminate NSR, CHF, and atrial fibrillation subjects [26]. Gridded Distribution Entropy differentiated healthy young people from healthy aged adults, as well as distinguishing healthy subjects from patients with coronary artery disease [29].

There are many papers in which more than one entropy definition is exploited. Attention, Dispersion, Distribution, and Phase entropy have been applied to HRV estimation, Chronic Chagas disease, and Cardiomyopathy detection [94]. Bubble and Phase entropy were used for coronary artery disease, sudden cardiac arrest, and congestive heart failure detection in [95], while Dispersion, Slope, Increment, and Phase entropy were used for myocardial infarction in [33]. Bubble Entropy and Dispersion Entropy have been utilized for HRV estimation on normal sinus rhythm, congestive heart failure, and coronary artery disease recordings [96]. Phase, Bubble, and Gridded Distribution Entropy have been employed in [28] for congestive heart failure and normal sinus rhythm recordings, while Attention and Bubble Entropy for the study of recordings of young and elderly subjects, congestive heart failure, and atrial fibrillation patients [30].

### 5.3. Articles on Cardiotocogram

The Cardiotocography signal monitors the fetal heart rate and uterine contractions during pregnancy and labor. It helps assess fetal well-being and detects potential complications related to oxygen levels and uterine activity. Bubble Entropy has been used both to evaluate the cord artery pH in labor [97] and to assess the well-being of the fetuses [98].

### 5.4. Articles on Photoplethysmogram

The Photoplethysmogram signal measures changes in blood volume using light absorption, typically through a sensor on the skin. It is commonly used for monitoring heart rate, oxygen saturation, and vascular health. Changes in its variability can indicate stress and potential underlying health issues. In [99], Attention, Dispersion, and Slope entropy were used to extract complexity features to discriminate between normal and cerebral infarction subjects, and Bubble Entropy was used for blood pressure estimation and stratification [100].

### 5.5. Articles on Electrohysterography

The examined entropy definitions have also been applied to signals acquired through Electrohysterography. The EHG signal is a measurement of electrical activity in the uterus, often used to assess uterine contractions during labor. It provides insights into the coordination and strength of uterine muscle activity. There are four papers referencing one of the examined definitions. Phase entropy has been used to analyze term and pre-term signals [101] and signals from low-risk pregnant subjects during parturition [102], while Dispersion Entropy and Bubble Entropy were used for pre-term birth prediction [103]. Bubble Entropy was employed to compare uterine myoelectrical activity between single and multiple gestation women [104]. Finally, Dispersion Entropy has been used to investigate term and pre-term births [105].

### 5.6. Articles on Electromyography

Electromyography measures the electrical activity of muscles. Variability in EMG is important because it reflects motor unit recruitment and neuromuscular control. Healthy variability indicates flexible and adaptive motor output, while reduced or excessive variability can signal neurological or muscular dysfunction, such as in Parkinson’s disease or spasticity. Bubble Entropy was used both to distinguish EMG fatigue and non-fatigue signals [106] and to investigate the neuromuscular system [107]. A classification between fatigue and non-fatigue subjects was also performed in [108], based on the extracted Phase entropy features. Slope entropy was used to classify EMG signals of patients with myopathy, neuropathy patients, and healthy subjects [18].

## 6. Discussion

Entropy is a measure of the system’s uncertainty used to distinguish between order and randomness. Beyond the examined definitions in this review, numerous other entropy measures have been proposed in the literature, since the field has been active for much more than a decade. Researchers have long been intrigued by the idea of measuring the uncertainty of systems, proposing novel entropy definitions. As a result, valuable metrics have been developed to further investigate the properties of a time series. A variety of research fields have adopted these measures in their studies, including physiological signal analysis.

To date, not only do we not know which entropy definition performs best, but we also know that the performance of each definition depends on the data set. This remains a long-standing and interesting problem for researchers to investigate. What they currently do is employ more than one definition and combine the results. With the widespread use of machine learning, this has become an easier task and a more straightforward choice.

However, we have to note that researchers usually select the most popular or well-established methods for their studies or for producing features for machine learning. They also do not justify their selections. It is true that they do not know a priori which method will perform best, and only after the computation can they assess their value. We hope that this review will help towards the direction of adding more entropy definitions in their experiments.

A main disadvantage of all entropy measures is the dependence on parameters. Both Approximate and Sample Entropy, for example, depend on the appropriate selection of two parameters: the embedding dimension (*m*) and the threshold distance (*r*), with the last being a real number. Values of *m* are practically limited to the range m=1…4, but *r* is a real parameter with an infinite domain set. Optimal estimation of these parameters is difficult or impossible, since they depend on the data set. Typical values, m=2,r=0.2, are almost always used as a confession that the problem is non-trivial. Parameter selection is a difficult problem for all entropy definitions. Researchers propose entropy measures that avoid proposing specific typical values for the parameters. Parameter estimation is still an open problem and an application-dependent issue.

There are some limitations of this work and potential biases. We used a single database, Scopus. Even though this was the most straightforward selection, it could narrow our research results. Then our subject-area filters could also limit the extent of the search. For example we already noted that we used the subject “Computer Science and Engineering” and the term “Biomedical” in the search criteria, as the umbrella of “Biomedical Engineering”, since the latter was not an option in the available search criteria of Scopus. We narrowed our search to articles written in English. Finally, as we stated before, we also have to mention here that we only included the original definitions and not their variants. For example we considered only Bubble Entropy [23,24] and not multiscale Bubble Entropy [109]. There is always the risk of missing preprints. On the other hand, we did not limit our search to journals only, but included conferences as well, as returned by the query to Scopus.

## 7. Conclusions

In this paper, we conducted a systematic and comprehensive review of recent entropy definitions proposed within the last decade (2015–2024). The PRISMA methodology was adopted as the most appropriate framework for conducting a systematic literature review. Using the Scopus database, we initially identified 516 papers related to physiological signals. After removing duplicates, this number was reduced to 447 and further refined to 213 by focusing exclusively on the Biomedical field. Ultimately, 92 papers that employed recent entropy definitions were included in our analysis.

We presented a table summarizing the total number of citations per entropy definition, along with the number of applications for each definition across various physiological signals. Dispersion Entropy had the highest number of citations (31 papers), followed by Bubble Entropy (25 papers) and Distribution Entropy (22 papers).

Additionally, we included a chart illustrating the distribution of the reviewed papers by publisher. IEEE accounted for the largest share (43 papers), followed by MDPI (16 papers).

A bar chart was also presented, showing the number of studies per physiological signal. The most frequently analyzed signals were EEG (43 papers) and ECG/HRV (41 papers). Other signals included, though with fewer references, were EHG (5), EMG (5), CTG (2), and PPG (2).

Finally, in the last section, each selected paper was individually reviewed. The methodology and objectives were documented, including the specific entropy definition applied and the physiological signal it was used on.

## Figures and Tables

**Table 1:** Description of symbols.

Symbol	Description
$x_{i}$	Sample from the time series
*N*	Length of the time series
$X=x_{1},x_{2},\ldots ,x_{N}$	Original time series
*m*	Embedding dimension
*d*	Time delay
$x_{i}^{m}=\left(x_{i},x_{i+d},\ldots ,x_{i+(m-1)d}\right)$	Embedded vector
$X^{m}=x_{1}^{m},x_{2}^{m},\ldots ,x_{N-m+1}^{m}$	Embedded series from *X*
$U=u_{1},u_{2},\ldots ,u_{N}$	Series of symbols
$U^{m}=u_{1}^{m},u_{2}^{m},\ldots u_{N-m+1}^{m}$	Embedded series of symbols

**Table 2:** Entropy definitions categorized by their conceptual families.

Definition Family	Definition
Embedding and Distance-Based	Range Entropy
	Cosine Similarity Entropy
	Diversity Entropy
	Distribution Entropy
Symbolic and Ordinal Pattern-Based	Increment Entropy
	Dispersion Entropy
	F-B Dispersion Entropy
	Slope Entropy
	Symbolic Dynamic Entropy
Complexity Estimation Based on Sorting Effort	Bubble Entropy
Multiscale and Hierarchical Definitions	Entropy of Entropy
Geometric or Phase-Space Definitions	Phase Entropy
	Gridded Distribution Entropy
Pattern-Detection Definitions	Attention Entropy

**Table 3:** Number of articles per method.

	As a Citation	In Title	In Keywords	In Abstract
Dispersion Entropy:	31	12	16	31
Bubble Entropy:	25	6	9	19
Distribution Entropy:	22	6	5	20
Increment Entropy:	8	2	5	8
Phase Entropy:	9	3	3	9
Slope Entropy:	6	2	1	4
F-B Dispersion Entropy:	4	1	1	3
Attention Entropy:	5	1	1	3
Gridded Distribution Entropy:	5	1	1	4
Range Entropy:	2	1	2	2
Entropy of Entropy:	3	1	-	3
Symbolic Dynamic Entropy:	2	-	-	1
Cosine Entropy:	2	-	-	1
Diversity Entropy: ^1^	-	-	-	-

^1^ Even though Diversity entropy had no citations, it is included, since it is a recently proposed definition.

**Table 4:** Number of articles per method and physiological signal.

	EEG	ECG/HRV	PPG	EHG	EMG	CTG
Dispersion Entropy:	20	10	1	2	-	-
Bubble Entropy:	8	10	1	2	2	2
Distribution Entropy:	5	15	-	-	1	-
Increment Entropy:	5	5	-	-	-	-
Phase Entropy:	2	4	2	-	1	-
Slope Entropy:	4	2	1	-	1	-
F-B Dispersion Entropy:	3	-	-	-	-	-
Attention Entropy:	1	2	-	-	-	-
Range Entropy:	2	-	-	-	-	-
Gridded Distribution Entropy:	2	2	-	-	-	-
Entropy of Entropy:	-	2	-	-	-	-
Symbolic Dynamic Entropy:	1	-	-	-	-	-
Cosine Entropy:	1	-	-	-	-	-
Diversity Entropy:	-	-	-	-	-	-
